# A proof-of-concept study for the use of a computerised avatar to embody the eating disorder voice in anorexia nervosa

**DOI:** 10.1007/s40519-022-01487-3

**Published:** 2022-10-22

**Authors:** Valentina Cardi, Thomas Ward, Viviana Aya, Chiara Calissano, Alistair Thompson, Janet Treasure

**Affiliations:** 1 Department of General Psychology, University of Padova, Via Venezia 8, 35131 Padua, Italy; 2 Department of Psychological Medicine, Institute of Psychiatry, Psychology and Neuroscience, King’s College London, London, UK; 3 Department of Psychology, Institute of Psychiatry, Psychology and Neuroscience, King’s College London, London, UK

**Keywords:** Anorexia nervosa, Avatar, Feasibility, Power, Voice

## Abstract

**Purpose:**

This study assessed (1) the experience of the eating disorder voice in people with anorexia nervosa or in remission, and (2) the feasibility of creating and interacting with a computerised representation (i.e., avatar) of this voice.

**Methods:**

Twenty-one individuals with anorexia nervosa and 18 individuals who were in remission participated in the study. They reported on the characteristics of their eating disorder voice and created a personalised avatar (a visual and auditory representation of the eating disorder voice), using a computerised software. Participants assessed closeness of match between the voice and the avatar, perceived distress and acceptability of re-exposure to the avatar.

**Results:**

Patients felt less powerful than their eating disorder voice and unable to disregard the voice's commands. The experience of the voice was associated with negative, as well as some positive emotions, reflecting the prototypical ambivalence towards the illness. Individuals in remission had an opposite pattern of responses. They attributed only negative emotions to the voice, felt more powerful than the voice, and able to disregard its commands. Overall participants reported that there was a good match between the voice and the sound of the avatar. Patients expressed willingness to repeat exposure to the avatar.

**Conclusion:**

Individuals with anorexia can create personalised digital avatars representing the eating disorder voice and are willing to engage therapeutically with the avatar. The next step is to test the feasibility of repeated exposure to the avatar to address the power and distress associated with the eating disorder voice.

**Level of evidence:**

Level III.

## Introduction

Anorexia nervosa is a psychiatric disorder characterised by persistent restriction of food intake and dangerously low body weight [1]. Patients often experience repetitive and persistent thoughts about the need to adhere to rigid rules about food restriction, weight and shape control, and anchor their self-value to the success in achieving this goal (e.g., [20]). Unhelpful cognitions (e.g., “I am nobody without my eating disorder”) and emotions (e.g., shame, guilt, sadness) related to the disorder are targeted to various degrees by currently available psychological treatments for anorexia nervosa, such as Cognitive-Behavioural Therapy, the Maudsley Model of Anorexia Nervosa Treatment for Adults and Focal Psychodynamic Psychotherapy. However, while these approaches tend to challenge the “content” of these beliefs, for example, with respect to “irrationality” and “unhelpfulness”, they often neglect the relationship that individuals have with these thoughts. This relationship is typically highly distressing for patients and their families and is characterised by the belief that the thoughts are uncontrollable and stronger than the individual’s willingness to challenge them (e.g., [23]).

A range of therapeutic approaches (e.g., relational, dialogical and narrative therapies) have been developed to target these unhelpful beliefs and the subordinate and powerless attitude that individuals have towards their negative thoughts, mindsets or critical parts of the self (e.g., [3, 11, 12]). These approaches often utilise externalisation techniques which enable individuals to engage in a dialogue with these other parts of the self, such as the “chairwork” technique [21]. These techniques encourage individuals to alternate between different “thinking positions” (for example, by changing sitting positions) to explore the origins, motivation and functions of the painful thoughts, and are aimed at building power, confidence and control over them [21]. Similarly, therapeutic approaches such as compassion-focussed therapy involve cultivating a “compassionate self/mind” which serves to counteract a more destructive part of the self, that is the “self-critic” [10].

The use of relational or dialogic approaches to psychopathology has gained momentum especially in the field of psychosis, where negative aspects of the self and critical inner speech can be experienced as voices (or auditory verbal hallucinations), through attribution to an external source [5, 14] or dissociated parts of the self (Waters et al. [25]). AVATAR therapy is a novel relational approach which involves creating a digital embodiment of the distressing voice (“the avatar”), which matches the acoustic experience and associated imagery. Face-to-face dialogues between the person and the avatar are facilitated by the therapist (using voice transformation software) and are aimed at gaining increased power and control over the abusive voice. A proof-of-concept study [17] and a fully powered randomised controlled study [6] have demonstrated the efficacy of AVATAR therapy in reducing the frequency, distress and power associated with the voices in psychosis with ongoing work focussed on optimisation and wider implementation [9].

The eating disorder voice is defined as “an internal voice experienced as second- or third-person commentary on shape, weight and eating, as well as the implications of these on self-worth” [2], page 348). The findings of a recent review on this phenomenon [2] indicated that patients frequently report the experience of the eating disorder voice (e.g., 94–96% of patients [20], and that the voice is typically perceived as “malevolent”, “omnipotent” and “powerful”, especially as the illness progresses [2]. Living with the eating disorder voice is experienced as a “battle” and associated with negative feelings; however, patients also report some ambivalence towards it, and can value it as a familiar companion [2]. Finally, the intensity of the eating disorder voice is associated with clinical severity, including weight and eating disorder symptoms (i.e., the stronger the voice, the worse the symptoms) [2]. While patients progress along the journey to recovery, they report changes in the content and relationship with the eating disorder voice [2]. In particular, they describe progressively gaining greater power over the voice [15]. These findings provide the rationale for targeting the eating disorder voice in patients with anorexia nervosa, with the overall goal of supporting individuals to gain power over the voice and reclaim an identity separate to the eating disorder.

The first goal of this study was to explore the characteristics of the eating disorder voice in anorexia nervosa. Considering the potential changes in the relationship with the voice through the recovery journey, the experience of the eating disorder voice was compared between patients (i.e., currently ill individuals), and a group who were in remission from the illness. The second goal was to create a visual and auditory representation of the eating disorder voice in the same two groups of participants, through the AVATAR therapy software. Closeness of visual and auditory match between the eating disorder voice and the avatar, perceived distress and acceptability of re-exposure to the avatar were assessed.

## Methods

### Participants

Thirty-nine female participants were included in this study: 21 individuals with a current DSM-5 diagnosis of anorexia nervosa and 18 individuals who were in remission. Participants were recruited from the South London and Maudsley NHS Foundation Trust and the Institute of Psychiatry Eating Disorders Unit’s volunteer database. Participants with anorexia nervosa had been diagnosed by a clinician and were receiving inpatient or outpatient eating disorder treatment at the time of their participation in the study. The group of participants in remission consisted of individuals who had a body mass index (BMI; kg/m^2^) of at least 18.5, reported no restriction and weight loss in the prior three months, and scored within 1 SD of age-matched community norms [19] on each of the Eating Disorder Examination Questionnaire’s subscales. Ethical approval was obtained from the UK London-Hampstead Research Ethics Sub-Committee (18/LO/0168). All participants provided written informed consent to take part in the study.

### Materials

Participants completed a baseline assessment consisting of:

A short demographic questionnaire, which included questions on ethnicity, age, years of education, civil status, psychiatry disorders and medication.

The Eating Disorder Examination Questionnaire (EDE-Q, [8], for the assessment of the eating disorder psychopathology.

Eating disorder voice assessment: five closed questions about the origin, beliefs and behavioural and emotional responses elicited by the voice. The questions were: “Do you identify the voice as internal or external?”; “Do you identify the content of the voice as a command or suggestion?”; “Who do you think is more powerful, yourself or the voice?”; “Most of the time, do you comply or resist/disregard the voice?”, and “Which of the following emotions does the voice elicit in you?; options were: anger, disgust, fear, happiness, joy, peace, relief, reassurance and sadness, and participants could select more than one. To gather additional details about the eating disorder voice, two open-ended questions were included. The first question asked participants to describe the main characteristics of the voice. The second question asked to provide details about the way in which the voice was helpful or unhelpful to them.

Avatar’s creation feedback: four visual analogue scales (VAS; ranging from 1 to 5) to assess the experience of creating a computerised representation (i.e., avatar) of the eating disorder voice. The questions were rated on a five-point Likert scale and were: (1) “To what extent did the avatar that you created match the sound of the eating disorder voice?”, (2) “To what extent did the avatar that you created match the image of the eating disorder voice?”; (3) “To what extent did you find it distressing to be exposed to the avatar?”; (4) “To what extent would you be willing to re-expose yourself to the avatar?”.

### Study design and procedure

This study was carried out in a single 60-min session. The session started with completion of the questionnaires. Then, a research assistant guided participants to use a computerised software to create a digital representation of the eating disorder voice (i.e., an avatar’s face). Participants were told to think about their eating disorder voice, including how it might look, and sound like. The software enabled participants to create the voice and face of a personalised avatar by selecting features from a range of pre-defined voices (e.g., tone, pitch), face (e.g., shape and colour of eyes, shape of nose and lips), and hairstyle features ([6], Leff, Williams, Huckvale, Arbuthnot and Leff [17]). Participants could select one feature at a time and change it until they were satisfied with it. Once they felt they had created a good enough visual representation of the avatar’s face, and that they had selected the appropriate voice features, the researcher asked participants to recall three common statements that the voice usually tells them and to choose the most distressing one to be spoken by the avatar (e.g., “you are ugly”, “you are a lazy pig”, “you are worthless”). Each participant was then exposed to the avatar’s face they created, which spoke the statement they had come up with (approximately 10 s). Following exposure to the avatar and the spoken message, participants were asked to complete the avatar’s creation feedback.

### Data analyses

Statistical analyses were carried out using SPSS version 24. Independent samples *t* tests and chi-square tests were used to compare sociodemographic data and EDE-Q scores between groups. Frequencies and percentages were described for the closed questions and the visual analogue scales. The circumplex model of emotions, a dimensional approach which classifies emotions based on arousal (low to high energy) and valence (displeasure to pleasure) [22] was used to describe the frequencies of emotions elicited by the avatar. Based on this approach, emotions were classified as (1) high arousal and negative valence (anger, disgust, fear); (2) high arousal and positive valence (happiness and joy); (3) low arousal and negative valence (sadness); and (4) low arousal and positive valence (peace, relief, reassurance).

Answers to the open questions were analysed using thematic analysis (Braun and Clark [4]) to identify, analyse and report the recurrent patterns produced in response to the questions [“themes”]. Data coding and analysis were undertaken by two independent raters, through an iterative process which involved reading of participants’ answers, identification of initial codes (i.e., sentence by sentence coding) and organisation of the codes into themes.

## Results

### Participants’ characteristics

Forty-one female participants were screened for eligibility. Two participants did not meet the inclusion criteria for the group in remission (i.e., they scored within more than 1 standard deviation of age-matched community norms). The final sample (*N* = 39) consisted of 21 individuals with a current DSM-5 diagnosis of anorexia nervosa, and 18 individuals in remission. Participants’ sociodemographic characteristics and differences between groups are described in Table 1.

**Table 1:** Demographic and clinical data for people with anorexia nervosa and individuals in remission

	Anorexia nervosa (*N* = 21)	Individuals in remission (*N* = 18)	Test and significance values
Age	23.1 (6.9)	25.3 (4.0)	*t*(37) = −1.18, *p* = 0.24
Years of education	16.0 (2.1)	18.9 (2.6)	*t*(37) = −3.90, *p* < 0.0001
With no partner	80.9%	72.2%	*X*^2^ (2) = 1.603, *p* = 0.45
Body mass index	15.5 (1.2)	22.3 (1.7)	*t*(37) = −14.41, *p* < 0.0001
Eating disorder examination questionnaire–restriction	4.3 (1.2)	0.7 (0.3)	*t*(37) = 12.56, *p* < 0.0001
Eating disorder examination questionnaire–eating concern	4.1 (1.2)	0.6 (0.3)	*t*(37) = 12.17, *p* < 0.0001
Eating disorder examination questionnaire– shape concern	4.5 (1.1)	1.1 (0.4)	*t*(37) = 12.07, *p* < 0.0001
Eating disorder examination questionnaire–weight concern	4.3 (1.2)	0.9 (0.4)	*t*(37) = 11.61, *p* < 0.0001
Eating disorder examination questionnaire–total	4.3 (1.1)	0.8 (0.3)	*t*(37) = 12.41, *p* < 0.0001

Data are expressed as means and standard deviations. Independent sample *t *test and *p* values are provided for the comparisons between groups

### Sociodemographic and clinical variables

The demographic and EDE-Q questionnaires were completed by all participants. Participants’ age range was 16–39 years. Those with a current diagnosis of anorexia nervosa had fewer years of education, a significantly lower BMI, and higher EDE-Q scores compared to those in remission. Of those with a current diagnosis of anorexia nervosa, 48% (*N* = 10) had a duration of illness equal or less than three years, 43% (*N* = 9) reported suffering from other psychiatric disorders besides the eating disorder, and 81% (*N* = 17) were taking psychiatric medication.

### Eating disorder voice assessment

Table 2 summarises the responses to the voice assessment interview. All participants identified their eating disorder voice as internal and human, with the exception of five participants in remission who described it as a non-human creature (e.g., as an animal/abstract figure). All participants with anorexia nervosa perceived the voice as a command, as something to obey, and as more powerful than the self. None of the individuals in remission reported that the voice was more powerful than the self, or that it was something to obey. A minority (*N* = 7) still identified it with a command.

**Table 2:** Assessment of the origin, beliefs, behavioural and emotional responses associated to the eating disorder voice in people with anorexia nervosa or in remission

	Anorexia nervosa (*N* = 21)	Individuals in remission (*N* = 18)
Voice perceived as human	100% (*N* = 21)	72% (N = 13)
Internal origin of voice	100% (*N* = 21)	100% (*N* = 18)
Voice content identified as a command	100% (*N* = 21)	39% (*N* = 7)
Voice perceived as more powerful than the self	100% (*N* = 21)	0% (*N* = 0)
Compliance to voice	100% (*N* = 21)	0% (*N* = 0)

Data are expressed as frequencies and percentages

All participants with anorexia nervosa reported negative emotions of anger, disgust, fear and sadness in relation to the voice. The majority of those in remission (*N* = 13) reported feelings of anger, and a minority also reported feelings of disgust (*N* = 7) or sadness (*N* = 4). Interestingly, while none of those in remission reported positive emotions in response to the eating disorder voice (regardless of arousal), the majority of those suffering from anorexia nervosa, reported feelings of relief and reassurance (*N* = 18 and *N* = 17, respectively). Table 3 describes the frequency of reporting each emotion in the two groups.

**Table 3:** Frequencies of emotions experienced in relation to the eating disorder voice by people with anorexia nervosa or in remission

Emotion category	Emotion	Anorexia nervosa (*N* = 21)	Individuals in remission (*N* = 18)
High arousal and negative valence	Anger	100% (*N* = 21)	72% (*N* = 13)
	Disgust	100% (*N* = 21)	39% (*N* = 7)
	Fear	100% (*N* = 21)	0% (*N* = 0)
High arousal and positive valence	Happiness	0% (*N* = 0)	0% (*N* = 0)
	Joy	0% (*N* = 0)	0% (*N* = 0)
Low arousal and positive valence	Peace	0% (*N* = 0)	0% (*N* = 0)
	Relief	86% (*N* = 18)	0% (*N* = 0)
	Reassurance	81% (*N* = 17)	0% (*N* = 0)
Low arousal and negative valence	Sadness	100% (*N* = 21)	22% (*N* = 4)

In response to the first open question (“how would you describe the voice, i.e., what are its main characteristics?”), participants across groups described the voice using similar negative terms, such as “harsh”, “critical” and “hostile”, “intrusive”, “constant” and “demanding”. With regards to the second open question (i.e., how the voice could be helpful or not helpful), both helpful and unhelpful aspects were highlighted by patients. They reported that the voice allowed them to cope with difficult emotions and situations, and that through it they experienced a sense of achievement or control, and connection. The unhelpful aspects of the voice included its negative impact on mental and physical health, social and family relationships, education and employment. Individuals in remission described similar unhelpful aspects to those with anorexia nervosa and interestingly, they could not identify helpful characteristics or functions of the eating disorder voice. The themes emerged from the answers to the two open questions are described in Table 4.

**Table 4:** Main themes emerging in response to the open questions about the main characteristics of the eating disorder voice and its helpfulness

Question	Group	Answers	Main themes	Examples
What are the main characteristics of the voice?	Patients = 21	Aggressive, hostile, hash, controlling, intrusive, constant, critical and demanding	Negative characteristics (All)	“Very hostile, critical and constant. It doesn’t seem to go away”
	In remission = 18			
Is the voice helpful to you in any way?	Patients = 21	Helpful to cope with difficult situations or emotions, provides a sense of companionship, achievement and control	Coping mechanism (*N* = 14) Companionship (*N* = 8) Sense of achievement (*N* = 8) Sense of control (*N* = 9)	“Cope with difficult situations and not to feel alone”
		Unhelpful in relation to health, relationships with family members, friends, meeting new people, going to Uni, finishing school year and finding/keeping a job	Mental and physical health (*N* = 11) Social and family relationships (*N* = 10) Education (*N* = 4) Employment (*N* = 1)	“To continue my education. I have to postpone going to Uni”
	In remission = 18	Unhelpful in relation to physical and mental health, family relationships, isolation, education, professional development and employment	Mental and physical health (*N* = 15) Social and family relationships (*N* = 12) Employment (*N* = 4) Education/professional development (*N* = 3)	“Not helpful at all. When I had anorexia I thought I was in control of things but later on I realised it was a false control”

### Avatar’s creation feedback

All participants across groups reported that there was a “very good” or “good” match between the sound of the avatar and the imagined sound of their internal voice. With regards to the image match, approximately half of those with anorexia nervosa (48%), and a third of individuals in remission (33%) rated it as “good”. Almost 90% of individuals with anorexia nervosa reported some level of distress in response to the exposure to the avatar (62% reported feeling “distressed” and 24% reported feeling “a little distressed”), whereas only 11% of those in remission reported feeling “a little distressed”. None of the participants across groups reported feeling “very distressed” and, importantly, all said they would be the willing to be re-exposed to their eating disorder avatar in the future.

## Discussion

This study is the first to test whether individuals with anorexia can create and interact meaningfully with personalised digital avatars which represent the eating disorder voice. The experience of the eating disorder voice and engagement with personalised avatars were compared between patients with anorexia nervosa and individuals in remission. When describing their eating disorder voice, currently ill individuals reported a different pattern of response compared to those in remission. In people in the acute stage of anorexia nervosa, the eating disorder voice was described as more powerful than the self and as something to be obeyed. The eating disorder voice elicited a range of negative emotions, but was also associated with a sense of relief and reassurance, reflecting the relational ambivalence towards the voice in this acute stage. With regards to voice function, participants with anorexia nervosa identified its role in coping with difficult emotions and enabling achievement and connection. Despite these positive functions, the disruptive impact of the eating disorder voice on their health and life was also identified. In contrast, all of those in remission reported feeling more powerful than the eating disorder voice and able to disregard its commands. They also highlighted a range of negative emotions, and no positive emotions associated to it. Interestingly, five individuals in remission described the voice as non-human, potentially indicating a helpful externalisation of the voice and differentiation from the self. These findings are in line with previous accounts of the eating disorder voice [2], and also highlight interesting overlaps between the experience of the voice among people with anorexia nervosa and psychosis. Examples of commonalities are the malevolence and power associated to the voice, and the sense of inferiority and negative affect experienced in the relationship with the voice [18]. As well as similarities, this study also indicates an expected difference with reference to the source of the voice, which is typically perceived as internally generated by people with eating disorders and external in origin by people with psychosis [26].

These observations suggest the potential of using the AVATAR therapy approach to target the distress and power associated with the eating disorder voice in anorexia nervosa. The first step of the AVATAR therapy approach is to create an avatar, which sounds and looks like the powerful and malevolent voice that individuals experience. This study found that it was feasible and acceptable to create an eating disorder avatar which elicited a range of distressing emotions commonly associated with the eating disorder voice. The elicitation of (and habituation to) “online” negative affect alongside a strong “sense of voice presence” (i.e., delivery of a valid simulation of the voice) are considered key components of AVATAR therapy [24], as well as exposure-based treatments more generally [7]. The finding that while participants experienced some level of distress, all expressed a willingness to be re-exposed to the avatar in the future is important evidence supporting the acceptability of this approach for people with anorexia nervosa.

This study represents a crucial first step in the application of AVATAR therapy in anorexia nervosa and suggests the potential therapeutic value of targeting self-empowerment through dialogue. These insights are informing an adaptation of AVATAR therapy for anorexia nervosa, which is currently being tested in a case series of patients.

## Strengths and limitations

The main strengths of this study are that it discusses the evidence for a promising therapeutic target in the treatment of anorexia nervosa, i.e., the eating disorder voice, and that it demonstrates the feasibility of using a novel approach to target it, i.e., the creation of an avatar to embody the voice. The inclusion of a sample of individuals in remission is an added strength of the study, because it broadens the understanding of the characteristics of the eating disorder voice through the recovery journey. The main limitations of this work are the relatively small sample size, although this is still in line with the recommendations for feasibility studies (e.g., Lancaster and Dodd [16]), and the lack of a standardised assessment of the eating disorder voice. At the time this study was conducted, only measures adapted from other clinical populations (psychosis) were available. More recently, the Experience of an Anorexia VoicE Questionnaire (EAVE-Q) has been developed to assess the experience of the eating disorder voice in anorexia nervosa [13].

## What is already known on this subject?

People with eating disorders acknowledge the experience of living with an eating disorder voice. They describe the voice as “malevolent”, “omnipotent” and “powerful”, especially as the illness progresses. The intensity of the voice is associated with the eating disorder psychopathology.

## What this study adds?

This study examined for the first time the feasibility of creating a visual and audio digital representation of the eating disorder voice (i.e., an avatar). It demonstrates that patients with anorexia nervosa can create avatars of the eating disorder voice and that they are willing to engage in a dialogue with them to regain power and control over the illness.

## Data Availability

The dataset generated and analysed for the current study is available from the corresponding author on reasonable request.
